# Lineage Analysis of Circulating *Trypanosoma cruzi* Parasites and Their Association with Clinical Forms of Chagas Disease in Bolivia

**DOI:** 10.1371/journal.pntd.0000687

**Published:** 2010-05-18

**Authors:** Ramona del Puerto, Juan Eiki Nishizawa, Mihoko Kikuchi, Naomi Iihoshi, Yelin Roca, Cinthia Avilas, Alberto Gianella, Javier Lora, Freddy Udalrico Gutierrez Velarde, Luis Alberto Renjel, Sachio Miura, Hiroo Higo, Norihiro Komiya, Koji Maemura, Kenji Hirayama

**Affiliations:** 1 Department of Immunogenetics, Institute of Tropical Medicine (NEKKEN), Nagasaki University, Nagasaki, Japan; 2 Clinica Siraní, Santa Cruz, Bolivia; 3 Center for International Collaboration Research, Nagasaki University, Nagasaki, Japan; 4 Centro Nacional de Enfermedades Tropicales, Santa Cruz, Bolivia; 5 Hospital Universitario Japonés, Santa Cruz, Bolivia; 6 Centro de Enfermedades Cardiovasculares (BIOCOR), Santa Cruz, Bolivia; 7 Department of Tropical Medicine and Parasitology, School of Medicine, Keio University, Tokyo, Japan; 8 Department of Parasitology, Graduate School of Medical Sciences, Kyushu University, Fukuoka, Japan; 9 Department of Cardiovascular Medicine, Nagasaki University Graduate School of Biomedical Science, Nagasaki, Japan; 10 Global COE Program, Nagasaki University, Nagasaki, Japan; Emory University, United States of America

## Abstract

**Background:**

The causative agent of Chagas disease, *Trypanosoma cruzi*, is divided into 6 Discrete Typing Units (DTU): Tc I, IIa, IIb, IIc, IId and IIe. In order to assess the relative pathogenicities of different DTUs, blood samples from three different clinical groups of chronic Chagas disease patients (indeterminate, cardiac, megacolon) from Bolivia were analyzed for their circulating parasites lineages using minicircle kinetoplast DNA polymorphism.

**Methods and Findings:**

Between 2000 and 2007, patients sent to the Centro Nacional de Enfermedades Tropicales for diagnosis of Chagas from clinics and hospitals in Santa Cruz, Bolivia, were assessed by serology, cardiology and gastro-intestinal examinations. Additionally, patients who underwent colonectomies due to Chagasic magacolon at the Hospital Universitario Japonés were also included. A total of 306 chronic Chagas patients were defined by their clinical types (81 with cardiopathy, 150 without cardiopathy, 100 with megacolon, 144 without megacolon, 164 with cardiopathy or megacolon, 73 indeterminate and 17 cases with both cardiopathy and megacolon). DNA was extracted from 10 ml of peripheral venous blood for PCR analysis. The kinetoplast minicircle DNA (kDNA) was amplified from 196 out of 306 samples (64.1%), of which 104 (53.3%) were Tc IId, 4 (2.0%) Tc I, 7 (3.6%) Tc IIb, 1 (0.5%) Tc IIe, 26 (13.3%) Tc I/IId, 1 (0.5%) Tc I/IIb/IId, 2 (1.0%) Tc IIb/d and 51 (25.9%) were unidentified. Of the 133 Tc IId samples, three different kDNA hypervariable region patterns were detected; Mn (49.6%), TPK like (48.9%) and Bug-like (1.5%). There was no significant association between Tc types and clinical manifestations of disease.

**Conclusions:**

None of the identified lineages or sublineages was significantly associated with any particular clinical manifestations in the chronic Chagas patients in Bolivia.

## Author Summary

Around 30–50% of *Trypanosoma cruzi* infections in Latin America cause chronic Chagas disease 10–30 years after the primary infection due to lack of effective treatment. The major clinical complications associated with chronic Chagas disease are cardiac myositis (leading to cardiac failure), and autonomous neuroplexus degeneration of the digestive tract that can cause megacolon or megaesophagus. Therefore, there are three major clinical forms of Chagas disease; cardiac, digestive and indeterminate (asymptomatic). The parasites, which can infect humans as well as other mammals, are transmitted by species of triatomines commonly found in the Americas. The parasite is divided in at least six discrete typing units: TcI, TcIIa–e. In humans, the TcI is mainly observed in Central America and northern parts of South America while the TcIIb/d/e is confined mainly to the southern cone of Latin America. We determined which DTU were prevalent in chronic patients in Bolivia, where the three clinical forms and several DTUs of the parasites are present, in order to determine whether there was a link between a particular parasite DTU and a particular clinical outcome. We found a vast majority of TcIId but its kDNA polymorphism showed no association with any of the clinical manifestations of chronic Chagas.

## Introduction

Despite concerted efforts to control Chagas disease in Bolivia, the prevalence of *Trypanosoma cruzi* remains high [1]. For example, one study has shown that in the village of Tarija, South Bolivia, the prevalence in pregnant women can reach up to 33.9% [2]. Furthermore, the vector infection rate of triatomines in the southern zone of Cochabamba, in the center of Bolivia, was found to be 79%, which translates to a very high infection risk for children in this area [3]. In addition, seroprevalence studies in Santa Cruz, in the east of Bolivia, recorded parasite exposure positive rates of 50% and concluded that there was a significant risk of infection via blood transfusion [4], [5], [6].

The chronic stage of Chagas disease may be categorized into three major clinical forms; those involving cardiopathy, digestive tract pathology (such as megacolon), and indeterminate (asymptomatic) forms [7], [8]. There appears to be geographical variation in the development of these clinical forms in Latin America, but in Bolivia all three clinical forms are observed [8], [9], [10], [11], [12].

This variation in pathological manifestation has been reported to be related to differences in the efficiency of the human host's immune response, such as ability to control parasitemia, the strength of inflammatory reactions, and the induction of autoimmune responses [13], [14], [15]. However, the host factors underlying the mechanisms of the outcome of the disease remain undetermined. Another possible factor that determines the clinical course of Chagas is the pathogenicity of *T. cruzi* itself. The genetic polymorphism of *T. cruzi* population may be related to its variability in pathogenicity. As there is no sexual stage in the parasite's life cycle (although some genetic exchange might occur) [16], the majority of parasites belong to clonal lineages. Population genetic studies of *T. cruzi*, carried out by zymodeme, ribosomal RNA, miniexon, and genomic DNA analyses, have revealed two primary phylogenetic lineages [17], [18], [19]. Within the last few years there has been an attempt to standardize the nomenclature. *Trypanosoma cruzi* is divided into 6 Discrete Typing Units (DTU) TcI, TcIIa, TcIIb, TcIIc, TcIId and TcIIe [20], [21], [22], [23], [24], [25]. Geographical and epidemiological studies showed that the distribution of TcI and TcII vary geographically. TcI is prevalent in the northern part of Brazil, Central and North America [26], [27], [28] while TcII is found predominantly in Southern cone countries of Latin America [29], [30]. In Bolivia, the TcIId was found to be the most common [30]. Furthermore, Virreira et al, 2006 [31] further sub-divided the TcIId based on patterns of minicircle Hypervariable Region (HVR), classifying three subdivisions as Mn-like, Bug-like and TPK-like.

In this study, in order to elucidate parasite factors that might influence the outcome of clinical disease, we have systematically classified our Bolivian patients according to their Chagas-related clinical symptoms (indeterminate, cardiopathy and megacolon) and determined the lineages of circulating parasites in their blood using PCR amplification.

## Materials and Methods

### Patients

Three hundred and six outpatients with chronic Chagas disease (144 men and 162 women, mean age 44.7 years) were recruited from Centro Nacional de Enfermedades Tropicales (CENETROP) (120 men and 132 women), and from inpatients and past patients at the Hospital Universitario Japonés (HUJ) (24 men and 30 women) in Santa Cruz, Bolivia. CENETROP was functioning as a National Center for the diagnosis of Chagas, and so patients were sent from outpatient clinics in Santa Cruz. Upon medical examination of patients, if serological tests (Indirect Haemagglutination test (IHA) and Indirect Immunofluorescence test (IIF) [1]) were positive, they were asked to participate in the study and informed consent was obtained. In order to collect a larger sample size of patients with megacolon, post-surgical operational megacolon patients with retrospective seropositivity were also recruited to the study following the same procedure as at CENETROP. As the majority of the patients were infected from childhood, their original birth places were recorded. Within 306 subjects, 203 were originally from Santa Cruz, 1 from Beni, 37 from Cochabamba, 6 from La Paz, 3 from Oruro, 33 from Chuquisaca, 10 from Potosi, 12 from Tarija, and 1 who did not born in Bolivia.

For all the CENETROP participants, Electrocardiogram (ECG) at CENETROP, and X-ray of colon following barium enema at Clinica Siraní, Santa Cruz, Bolivia were performed. For the HUJ past patients with megacolon, we retrospectively checked their ECG abnormalities, macroscopic observation of colon pathology and serological tests.

ECG examination was performed using an automated ECG analyzer (Nihon-denshi, Tokyo, Japan) and ECG abnormalities were diagnosed base on Minnesota Code Criteria [32], [33] (Table 1). All the ECG records were checked and confirmed independently by two cardiologists (AG and LAR). Colon X-ray with barium enema examination was performed for the detection of megacolon as previously described [34]. No achalasia like symptoms was observed in any patients and barium enhanced X-ray examination for megaesophagus was not performed. ECG and Barium enema X-ray examinations were performed for all participants except for the post operational patients with megacolon. To exclude the possibility of including an asymptomatic person as an indeterminate form who did not have enough time to develop complications, asymptomatic patients who were less than 30 years old were excluded from the study.

**Table 1:** ECG abnormalities observed in chronic Chagas patients.

ECG abnormality	N = 64	ECG abnormality with megacolon	N = 17
cRBBB	12	MI	1
cRBBB, LAFB	1	iRBBB	2
cRBBB, LVH	1	cRBBB	3
cRBBB, SB	2	cRBBB, LAFB	3
iRBBB	9	sMI	1
iRBBB, SB	1	RVCD	1
AVB-1st	3	LAE, SB	1
AVB-2nd	1	PAC	1
MI	1	Short PR interval	1
sMI, RAD	1	cLBBB	1
iMI	1	LAFB, RVCD	1
aMI	2	AF	1
aMI, LAFB,	1		
asMI, LAFB	1		
SB	3		
ST	1		
LAD	5		
LAD, RVCD	2		
SAR	1		
ICD	2		
cLBBB	3		
LAE	1		
RVCD	7		
LVH	1		
T wave abnormalities	1		

cRBBB; complete right bundle branch block, LAFB; left anterior fascicular block, LVH; left ventricular hypertrophy, SB; sinus bradycardia, iRBBB; incomplete right bundle branch block, AVB-1^st^; first degree AV block, AVB-2^nd^; second degree AV block, MI; myocardial infarction, sMI; septal myocardial infarction, RAD; right axis deviation, iMI; inferior myocardial infarction, aMI; anterior myocardial infarction, asMI; anteroseptal myocardial infarction, ST; sinus tachycardia, RVCD; right ventricular conduction delay, SAR; sinus arrhythmia, ICD; intraventricular conduction delay, cLBBB ;complete left bundle branch block, LAD; left axis deviation, LAE; left atrial enlargement, PAC; premature atrial contraction, AF; atrial fibrillation.

The experimental protocol was approved by the Institutional Ethical Review Committee at the Institute of Tropical Medicine, Nagasaki University (No. 0210170018) and at the Centro Nacional de Enfermedades Tropicales (CENETROP). Written informed consent was obtained from all subjects.

### Blood culture, DNA extraction and PCR conditions

Three ml of heparinized blood were cultured in 7 ml of liver infusion tryptose (LIT) Becton Dickinson, MA,USA) medium supplemented with 10% Fetal Calf Serum (Invitrogen, CA, USA) and 0.05% Hemin (Sigma-Aldrich Inc., St. Louis, USA) for more than 3 months and, microscopically checked for the parasite growth, as previously described [35].

Genomic DNA was extracted from 10 ml of whole blood containing 10 mM ethylene diamine tetra acetic acid (EDTA) using a DNA extraction kit (QIAGEN GmbH, FRG) and was stored at −20°C.

PCR amplification was performed with two primer sets to detect *T. cruzi* genomic and mitochondrial DNA: Tcz1 (CGAGCTCTTGCCCACACGGGTGCT)/Tcz2 (CCTCCAAGCAGCGGATAGTTCAGG) for detection of genomic satellite DNA [36], 121 (AAATAATGTACGGGKGAGATGCATGA)/122 (GGTTCGATTGGGGTTGGTGTAATATA) for minicircle kDNA [30]. PCR was carried out in a total volume of 30 µl containing 1× buffer, 2 mM MgCl_2_, 0.2 mM dNTPs, and 1 µM each primer, 0.15 units of *Taq* polymerase (Takara Bio INC, JPN) and 150 ng of sample DNA. The PCR condition for Tcz1/Tcz2 and 121/122 were performed as described elsewhere [37], [38].

### DTUs analysis

Analysis of the lineage of *T. cruzi* was carried out by a sequence- specific oligonucleotide probe (SSOP) method [39]. Briefly, 2 µl of the amplified kinetoplast minicircle hypervariable region (kDNA) was blotted on positively-charged nylon membranes (Roche Diagnostics K.K, CH) and were UV- crosslinked for fixation after alkaline treatment. Ten micromole of the biotin labeled HVR domain of TcI or TcII (b, d, e) SSOPs were hybridized on a 6 cm×8 cm square membrane containing sample dots in 10 ml of hybridization buffer containing 50 mM Tris-HCl pH8, 0.3 M Tetramethylammonium Chloride (TMAC), 2mM EDTA, 5× Denhart's Solution (100 ml of 2% PVP, 2% Ficoll-400 and 2g of BSA), 0.1%SDS and 1 mg sonicated salmon sperm DNA at 56°C for 3 hours. Detection was performed by the Southern Light CSPD (TROPIX Inc. Bedford MA, USA) chemiluminescence method. The image was captured by Luminescent Image Analyzer, Image Reader LAS-4000 mini (Fujifilm K. K, Japan).

For the detection of TcI parasites, biotin labeled whole PCR fragment of kDNA-HVR of the strain Ab4-1 (TcI) was used for a hybridization probe, as previously described [39], [40]. For TcII parasites, six probes were prepared for TcIIb and one for TcIIe. Because TcIIa and TcIIc have been rarely observed in human patients, probes specific to these types were not used [41]. BLAST search allowed the construction of 6 oligonucleotides with high homology for TcIIb kDNA-HVR. The accession numbers correspond to the sequences in which the probes were designed. Oligo A (GTA GTT TAA GAT AAT ATC ATG T) (accession numbers: AJ747981, AJ747979, AJ747978), Oligo B (ATA GTT AAT TAT AGA ATA TTC TG) (accession number: AJ747983), Oligo C (TGA TAA CGT ATG TAT TAT GTT GA) (accession number: AJ747975), Oligo D (TAT AAT TAT GTA TAT CTT AAT GT) (accession number: AJ747977), Oligo E (TAT ATA TCT GAG TTA CTG TTG) (accession number: AJ747080, AJ747976) and Oligo F (TAT GGA GAC ATA GAA TTG AGT A) (accession number: AJ747995, AJ747994, AJ747992, AJ747991, AJ747990). BLAST search allowed the construction of Oligo H (TAA GAC ATG ACA TAA TAC AAT) from a sequence with high homology for TcIIe kDNA-HVR (accession numbers: AJ748068, AJ748061 and AJ748053). Detection of TcIId was performed with the Oli 1 and Oli 2 probes previously described [31]. The hybridization patterns obtained by the combination of those probes, Oli1+/Oli2−, Oli1−/Oli2+ and Oli+/Oli2+, were used for classification of the TcIId subtypes, Bug-like, Mn-Like and TPK-like respectively.

The strains PH 1 (TcIIa), PH 2 (TcIIa) [42] were used as negative controls for the probes. The strains Bernice (TcIIb), Tul-L (TcIIb), Y (TcIIb) and Ab-3-8 (TcIId) [16] were used as positive controls. The hybridization profiles obtained with the probes are presented supplementary data (Figure S1).

### Statistical analysis

Two-tailed Chi square tests were used to determine the significance of associations between parasite type and clinical manifestations of disease. Contingency tables were used to determine the odds ratio (OR) and a 95% confidence interval (CI) by Woolf's formula.

## Results and Discussion

### Clinical identification of seropositive chronic patients

All patients (n = 306) were serologically positive. Out of 231 ECG examinees, 81 patients (35.1%) had cardiac abnormalities as identified by electrocardiogram (38 men and 43 women, mean age 41.0 years, age range 22–72). One hundred patients had megacolon as determined by barium enema X-ray examination (n = 45) or by the macroscopic observation at the surgical operation (n = 55) (50 men and 50 women, mean age 51.3 years, age range 17–89). Out of 189 barium enema examinees, 45 (23.8%) were diagnosed megacolon and 21 patients (46.7%) out of the 45 megacolon patients showed elongation of the sigmoid colon [43]. Out of 114 patients who had both ECG and barium enema X-ray examinations, 73 patients (64%) without any complications after the above mentioned examinations were deemed indeterminate cases (34 men and 39 women, mean age 42.3 years, age range 29–65) (Table 2).

**Table 2:** Clinical manifestations of the subjects and their circulating parasite detection by PCR.

ECG Abnormality	Megacolon	No. of patients	Male	Female	Age			121/122 PCR(+)^a^ (%)
					Mean	±	SD	
**−**	**−**	73	34	39	42.3	±	8.5	57.5
**−**	NE^b^	41	16	25	42.9	±	9.6	61.0
**+**	NE	21	10	11	40.6	±	10.4	52.4
**+**	**−**	43	22	21	38.5	±	9.8	72.1
**+**	**+**	17	6	11	47.9	±	14.1	52.9
**−**	**+**	36	17	19	47.1	±	12.0	55.6
NE	**+**	47	27	20	55.6	±	17.0	80.9
NE	**−**	28	12	16	42.5	±	7.4	71.4
**Total**		**306**	**144**	**162**	**44.7**	**±**	**12.4**	**64.1**

a Amplification of the minicircle kinetoplast hypervariable region for further lineages analysis.

b Not Examined: The subjects had no test of ECG or colon enema X ray.

### Detection of *T. cruzi* DNA from patients' peripheral blood by species specific primers

In acute Chagas disease, detection of parasite DNA is feasible due to high parasitemia, whereas in Chronic Chagas it is more difficult because of the generally lower parasitemia [44]. Using kDNA PCR products, we could detect circulating parasite genomic DNA from 64.1% of the samples and could analyze in detail the lineages of 47.4% of the 306 seropositive chronic patients as shown in Table 2. Tcz1/Tcz2 primer set for genomic satellite DNA detected 77.8% whereas the 121/122 primer that target the minicircle kDNA showed a lower sensitivity.

Three ml of blood samples were cultured from all the 306 seropositive samples, however, only two isolates were obtained after 2–3 months culture. These two isolates from a 50 year old male and a 52 year old female were named SCBOL1 and SCBOL2 respectively. Both these parasites were determined to be TcIId.

No significant deviation between the PCR negative samples and the categorized form of the disease was observed.

### Characterization of *T. cruzi* DTU I and II sublineages by minicircle kDNA HVR analysis

The minicircle kinetoplast is present at about 5000 copies per parasite and each minicircle has four hypervariable regions that correspond to families of specific DTU. These sequences were targeted for detection of clones of TcI and TcIId in previous work [30]. In this work, minicircle kDNA amplification allowed the identification of 64.1% (196 out of 306) of the seropositive chronic Chagas patients. Out of those 196 samples, 133 (68.0%) were identified as TcIId, 31 (15.8%) TcI, 10 (5.1%) TcIIb and 1 (0.5%) TcIIe (Table 3). Double or triple infection with Tc I/IId (26 out of 196), Tc I/IIb/IId (1 out of 196) or Tc IIb/IId (2 out of 196) was also detected. The remaining undefined samples (26.0%) could be due to the limitation of detection by the probes. It is possible that these clones may have a unique, as yet undescribed genotype which was not detected by the probes used in this study.

**Table 3:** Clinical manifestations and identified DTUs.

ECG abnormality	Mega- colon	Examined Number	TcI n (%)	TcIIb n (%)	Tc I/IId n (%)	Tc I/IIb/IId n (%)	Tc IIb/IId n (%)	TcIId n (%)	TcIIe n (%)	UND^a^ n (%)
**−**	**−**	42	0 (0.0)	1 (2.4)	8 (19.0)	0 (0.0)	0 (0.0)	24 (57.1)	0 (0.0)	9 (21.4)
**−**	NE^b^	25	0 (0.0)	0 (0.0)	1 (4.0)	0 (0.0)	0 (0.0)	20 (80.0)	0 (0.0)	4 (16.0)
**+**	NE	11	0 (0.0)	0 (0.0)	2 (16.7)	0 (0.0)	0 (0.0)	6 (54.3)	0 (0.0)	3 (25.0)
**+**	**−**	31	0 (0.0)	2 (6.3)	4 (12.5)	0 (0.0)	0 (0.0)	14 (43.8)	1 (3.1)	10 (32.3)
**+**	**+**	8	0 (0.0)	0 (0.0)	0 (0.0)	0 (0.0)	1 (12.5)	4 (50.0)	0 (0.0)	4 (44.4)
**−**	**+**	20	0 (0.0)	0 (0.0)	3 (15.0)	0 (0.0)	1 (5.0)	11 (55.0)	0 (0.0)	5 (25.0)
NE	**+**	38	4 (10.5)	3 (7.9)	4 (10.5)	1 (2.6)	0 (0.0)	17 (44.7)	0 (0.0)	9 (23.7)
NE	**−**	20	0 (0.0)	1 (5.0)	4 (20.0)	0 (0.0)	0 (0.0)	8 (40.0)	0 (0.0)	7 (35.0)
**Total**		**196**	**4 (2.0)**	**7 (3.6)**	**26 (13.3)**	**1 (0.5)**	**2 (1.0)**	**104 (53.1)**	**1 (0.5)**	**51 (26.0)**

a Unidentified Tc, those amplified minicircle DNA did not hybridize with any of the probes.

b Not Examined: The subjects had no test of ECG or colon enema X ray.

There was no significant difference between those TcI, IIb, IId and IIe distribution in the clinical groups, as shown in Table 3. However, these data partially support previous reports that TcI is predominantly observed in triatomines but not in elderly humans and that Tc IId predominantly infects humans in Bolivia [29], [30], [45] and in Argentina [44].

### TcIId subtype

Among the 133 TcIId samples that were positive with either Oli1 and/or Oli2 TcIId probes, 49.6% were Mn-like (Oli1−/Oli2+), 48.9% were TPK1-like (Oli1+/Oli2+) and 1.5% were Bug-like (Oli1+/Oli2−) (Figure S1). For the analysis of TcIId, the method reported by Virreira et al, 2006 using kDNA polymorphism was followed [31]. They observed that the three subtypes of TcIId, TPK1-like, Bug-like and Mn-like were almost equally distributed in 39 cord blood samples collected in Cochabamba and Tarija, Bolivia. While in our study, only two samples (1.5%) of the Bug-like type were detected as shown in Table 4. The reason for the low frequency of the bug-like in the present study may be due to the geographical difference between central and south in Bolivia. Another reason may involve the origin of blood samples; in the previous study [31], samples were from new born cord blood from infected mothers, whereas in the present study blood was obtained from adult patients with chronic infections.

**Table 4:** No association between clinical manifestations and major DTUs observed.

Patients group	No. of samples					TcIId subgroup			
		TcI (%)		TcIId (%)		Mn (%)		TPK (%)	
ECG (+)	35	6	(17.6)	31	(91.4)	17	(54.8)	13	(41.9)
ECG (−)	69	12	(17.4)	68	(98.6)	31	(45.6)	36	(52.9)
Megacolon (+)	49	12	(24.5)	42	(85.7)	18	(42.9)	22	(52.4)
Megacolon (−)	67	16	(23.9)	62	(92.5)	37	(59.7)	25	(40.3)
ECG (+) and/or Megacolon (+)	78	18	(22.8)	68	(87.2)	32	(47.5)	34	(50.0)
ECG (−) and Megacolon (−)	33	8	(24.2)	32	(97.6)	17	(53.1)	15	(46.9)

ECG (+): ECG abnormality was observed as described in Table 1. ECG (−): ECG examination detected no abnormality. Megacolon (+): Megacolon was observed by the X-ray examination after barium enema. Megacolon (−): Normal colon X-ray after barium enema. No significant statistical association was observed in any comparison.

Our results imply that the clinical manifestations of chronic Chagas disease (defined as cardiopathology, megacolon or indeterminate), are not correlated with parasite lineage or subgroup types of TcIId (Table 4). It has been proposed that a genetic mutation occurred following the divergence of the subgroups of TcIId that altered parasite pathogenicity. Given this, it is important that further parasite genetic characterization and association studies with clinical pathology are carried out. Several important genes that may be involved in the invasion, proliferation and immune evasion of these parasites have been identified [46], [47], and these would be obvious candidate genes for pathogenicity association studies.

The first paper that suggested differences in clinical spectrum by strain type was given by Miles et al that a markedly contrast between the main zymodemes observed in Venezuela (Z1 = TcI) and Brazil (Z2 = TcII) could explain the absence and presence of gastrointestinal Chagas respectively in the regions [48]. The lack of association between DTU and clinical picture in the present study can be explained by the reason that there was not enough heterogeneity in the genetic makeup of the parasites in this geographical area to provide the statistical power to demonstrate a difference. Therefore our study further supports the hypothesis that large-scale differences in clinical manifestations (presence or absence of GI megasyndromes) may be due to large genetic differences (Type I vs II, southern Brazil vs Venezuela) but that within a geographic area where one or 2 DTUs predominate as observed here, the differences are due to host factors.

In conclusion, the TcIId lineage was shown to be involved in the pathogenesis of a variety of clinical manifestations of chronic Chagas disease in Bolivia, but the subtypes of TcIId were not associated with any of the clinical forms.

## Supporting Information

Figure S1 Ten hybridization membranes using different probes for detection of DTU lineage, sublineage and subgroup are shown. The probes used here were as follows; A: Oli 1 (DTU IId), B: Oli2 (DTU IId), C–H: probes A, B, C, D, E, F (DTU IIb), I: probe H (DTU IIe) and J: DTU I.
(0.30 MB DOC)

## References

[1] WHO (2002). Control of Chagas disease: second report of a WHO expert committee.. Tech WHO.

[2] Brutus L, Schneider D, Postigo J, Romero M, Santalla J (2008). Congenital Chagas disease: Diagnostic and clinical aspects in ana rea without vectorial transmisión, Bermejo, Bolivia.. Acta Trop.

[3] Medrano-Mercado N, Ugarte-Fernandez R, Butron V, Uber-Busek S, Guerra HL (2008). Urban transmission of Chagas disease in Cochabamba, Bolivia.. Mem Inst Oswaldo Cruz.

[4] Schmunis GA, Zicker F, Pinheiro F, Brandling-Bennett D (1998). Risk for Transfusion-transmitted infectious diseases in Central and South America.. Emerg Infect Dis.

[5] Schmunis GA, Rodriguez G, Coenen J, Bellorin EG, Gianella A (2008). Prevention of blood-borne diseases in Bolivia, 1993–2002.. Am J Trop Med Hyg.

[6] Landivar WH, Nakasa T, Tachibana H, Paz KC, Tateno S (1992). Seropositivity to *Trypanosoma cruzi* in blood donors in Santa Cruz, Bolivia.. J Infect Dis.

[7] Prata A (2001). Clinical and epidemiological aspects of Chagas disease.. Lancet Infect Dis.

[8] Breniere SF, Carrasco R, Revollo S, Aparicio G, Desjeux P (1989). Chagas' disease in Bolivia: clinical and epidemiological features and zymodeme variability of Trypanosoma cruzi strains isolated from patients.. Am J Trop Med Hyg.

[9] Cabral HR, Glocker TM, Novak IT, Krainbuhl VA (1999). The esophagus in patients with Chagas disease in Cordoba, Argentine. Histologico-immunohistochemical, and evacuation time. Review.. Rev Fac Cien Med Univ Nac Cordoba.

[10] Carod-Artal FJ (2006). Enfermedad de Chagas e ictus.. Neurologia.

[11] Aguilar VHM, Abad-Franch F, Racines VJ, Paucar CA (1999). Epidemiology of Chagas disease in Ecuador. A brief review.. Mem Inst Oswaldo Cruz.

[12] Freitas JM, Andrade LO, Pires SF, Lima R, Chiari E (2009). The MHC gene region of murine hosts influences the differential tissue tropism of infecting Trypanosoma cruzi strains.. PLoS One.

[13] Marin-Neto JA, Cunha-Neto E, Maciel BC, Simoes MV (2007). Pathogenesis of chronic Chagas heart disease.. Circulation.

[14] Arce-Fonseca M, Ballinas-Verdugo MA, Reyes PA, Aranda-Fraustro A, Monteon VM (2005). Autoantibodies to human heart conduction system in Chagas' disease.. Vector Borne Zoonotic Dis.

[15] Manoel-Caetano Fda S, Silva AE (2007). Implications of genetic variability of *Trypanosoma cruzi* for the pathogenesis of Chagas disease.. Cad Saude Publica.

[16] Higo H, Miura S, Horio M, Mimori T, Hamano S (2004). Genotypic variation among lineages of *Trypanosoma cruzi* and its geographic aspects.. Parasitol Int.

[17] Miles MA, Souza A, Povoa M, Shaw JJ, Lainson R (1978). Isozymic heterogeneity of *Trypanosoma cruzi* in the first autochthonous patients with Chagas' disease in Amazonian Brazil.. Nature.

[18] Souto RP, Fernandes O, Macedo AM, Campbell DA, Zingales B (1996). DNA markers define two major phylogenetic lineages of *Trypanosoma cruzi*.. Mol Biochem Parasitol.

[19] Fernandes O, Mangia RH, Lisboa CV, Pinho AP, Morel CM (1999). The complexity of the sylvatic cycle of *Trypanosoma cruzi* in Rio de Janeiro state (Brazil) revealed by the non-transcribed spacer of the mini-exon gene.. Parasitology.

[20] Tibayrenc M (1995). Population genetics of parasitic protozoa and other microorganisms.. Adv Parasitol.

[21] Tibayrenc M (1998). Genetic epidemiology of parasitic protozoa and other infectious agents: the need for an integrated approach.. Int J Parasitol.

[22] Brisse S, Barnabe C, Tibayrenc M (2000). Identification of six *Trypanosoma cruzi* phylogenetic lineages by random amplified polymorphic DNA and multilocus enzyme electrophoresis.. Int J Parasitol.

[23] Brisse S, Verhoef J, Tibayrenc M (2001). Characterization of large and small subunit rRNA and mini-exon genes further supports the distinction of six *Trypanosoma cruzi* lineages.. Int J Parasitol.

[24] Zingales B, Souto RP, Mangia RH, Lisboa CV, Campbell DA (1998). Molecular epidemiology of American trypanosomiasis in Brazil based on dimorphisms of rRNA and mini-exon gene sequences.. Int J Parasitol.

[25] Westenberger SJ, Cerqueira GC, El-Sayed NM, Zingales B, Campbell D (2006). *Trypanosoma cruzi* mitochondrial maxicircles display species- and strain-specific variation and a conserved element in the non-coding region.. BMC Genomics.

[26] Anez N, Crisante G, da Silva FM, Rojas A, Carrasco H (2004). Predominance of lineage I among *Trypanosoma cruzi* isolates from Venezuelan patients with different clinical profiles of acute Chagas' disease.. Trop Med Int Health.

[27] Ruiz-Sanchez R, Leon MP, Matta V, Reyes PA, Lopez R (2005). *Trypanosoma cruzi* isolates from Mexican and Guatemalan acute and chronic chagasic cardiopathy patients belong to *Trypanosoma cruzi* I.. Mem Inst Oswaldo Cruz.

[28] Zingales B, Stolf BS, Souto RP, Fernandes O, Briones MR (1999). Epidemiology, biochemistry and evolution of *Trypanosoma cruzi* lineages based on ribosomal RNA sequences.. Mem Inst Oswaldo Cruz.

[29] Breniere SF, Bosseno MF, Telleria J, Bastrenta B, Yacsik N (1998). Different behavior of two *Trypanosoma cruzi* major clones: transmission and circulation in young Bolivian patients.. Exp Parasitol.

[30] Breniere SF, Bosseno MF, Noireau F, Yacsik N, Liegeard P (2002). Integrate study of a Bolivian population infected by *Trypanosoma cruzi*, the agent of Chagas disease.. Mem Inst Oswaldo Cruz.

[31] Virreira M, Alonso-Vega C, Solano M, Jijena J, Brutus L (2006). Congenital Chagas disease in Bolivia is not associated with DNA polymorphism of *Trypanosoma cruzi*.. Am J Trop Med Hyg.

[32] Blackburn H, Keys A, Simonson E, Rautaharju P, Punsar S (1960). The electrocardiogram in population studies. A classification system.. Circulation.

[33] Blum JA, Zellweger MJ, Burri C, Hatz C (2008). Cardiac involvement in African and American Trypanosomiasis.. Lancet Infect Dis.

[34] Earlam RJ (1972). Gastrointestinal aspects of Chagas' disease.. Am J Dig Dis.

[35] Isola EL, Lammel EM (1989). *Trypanosoma cruzi*: conditions required to improve metacyclic differentiation in axenic culture. Redruello M.. Rev Argent Microbiol.

[36] Moser DR, Cook GA, Ochs DE, Bailey CP, McKane MR (1989). Detection of *Trypanosoma congolense* and *Trypanosoma brucei* subspecies by DNA amplification using the polymerase chain reaction.. Parasitology.

[37] Wincker P, Britto C, Pereira JB, Cardoso MA, Oelemann W (1994). Use of a simplified polymerase chain reaction procedure to detect *Trypanosoma cruzi* in blood samples from chronic chagasic patients in a rural endemic area.. Am J Trop Med Hyg.

[38] Virreira M, Torrico F, Truyens C, Alonso-Vega C, Solano M (2003). Comparison of polymerase chain reaction methods for reliable and easy detection of congenital *Trypanosoma cruzi* infection.. Am J Trop Med Hyg.

[39] Yoshida M, Kimura A, Numano F, Sasazuki T (1992). Polymerase-chain-reaction-based analysis of polymorphism in the HLA-B gene.. Hum Immunol.

[40] Veas F, Breniere SF, Cuny G, Brengues C, Solari A (1991). General procedure to construct highly specific kDNA probes for clones of *Trypanosoma cruzi* for sensitive detection by polymerase chain reaction.. Cell Mol Biol.

[41] Barnabe C, Brisse S, Tibayrenc M (2000). Population structure and genetic typing of *Trypanosoma cruzi*, the agent of Chagas disease: a multilocus enzyme electrophoresis approach.. Parasitology.

[42] Higo H, Miura S, Agatsuma T, Mimori T, Yanagi T (2007). Identification of *Trypanosoma cruzi* sublineages by the simple method of single-stranded conformation DNA polymorphism (SSCP).. Parasitol Res.

[43] Garcia RL, Matos BM, Féres O, Rocha JJ (2008). Surgical treatment of Chagas megacolon. Critical analysis of outcome in operative methods.. Acta Cir Bras.

[44] Bosseno MF, Torrico F, Telleria J, Noireau F, Breniere SF (1995). Reaccion de polimerizacion en cadena: Deteccion y caracterizacion de cepas de *Trypanosoma cruzi* en ninos Chagasicos.. Medicina.

[45] Burgos JM, Altech J, Bisio M, Duffy T, Valadares HM (2007). Direct molecular profiling of minicircle signature and lineages of *Trypanosoma cruzi* bloodstream populations causing congenital Chagas disease.. Int J Parasitol.

[46] Songthamwat D, Kajihara K, Kikuchi M, Uemura H, Tran SP (2007). Structure and expression of three gp82 gene subfamilies of *Trypanosoma cruzi*.. Parasitol Int.

[47] Rubin de Celis SS, Uemura H, Yoshida N, Schenkman S (2006). Expression of trypomastigote trans-sialidase in metacyclic forms of *Trypanosoma cruzi* increases parasite escape from its parasitophorous vacuole.. Cell Microbiol.

[48] Miles MA, Cedillos RA, Povoa MM, de Souza AA, Prata A (1981). Do radically dissimilar *Trypanosoma cruzi* strains (zymodemes) cause Venezuelan and Brazilian forms of Chagas' disease?. Lancet.

