# Supplementary figures and images for: Lineage Analysis of Circulating Trypanosoma cruzi Parasites and Their Association with Clinical Forms of Chagas Disease in Bolivia
Source: PLoS Negl Trop Dis. 2010 May 18;4(5):e687. doi: 10.1371/journal.pntd.0000687 (PMC2872639; doi:10.1371/journal.pntd.0000687)

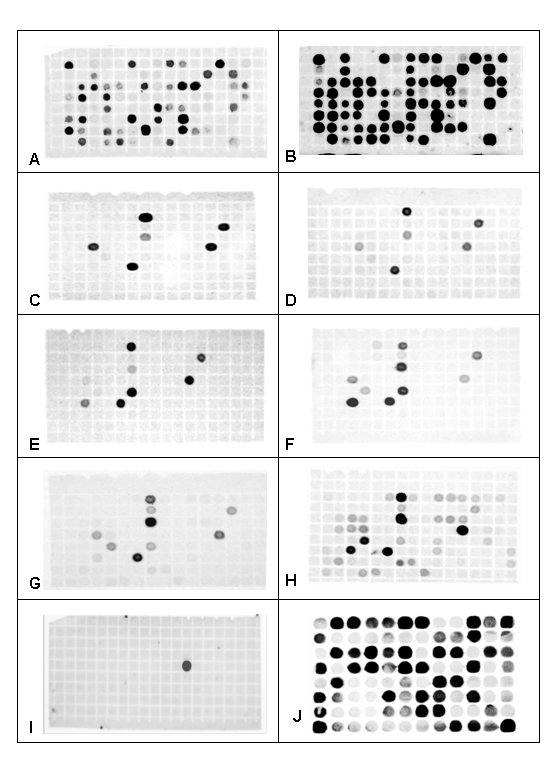


Supplement figure 1. Hybridization profiles with the probes.

Supplement: Figure S1 — Ten hybridization membranes using different probes for detection of DTU lineage, sublineage and subgroup are shown. The probes used here were as follows; A: Oli 1 (DTU IId), B: Oli2 (DTU IId), C–H: probes A, B, C, D, E, F (DTU IIb), I: probe H (DTU IIe) and J: DTU I. (0.30 MB DOC) [file pntd.0000687.s001.doc]
